# Supplementary material for: The systemic lupus erythematosus-associated NCF190H allele synergizes with viral infection to cause mouse lupus but also limits virus spread
Source: Nat Commun. 2025 Feb 13;16:1593. doi: 10.1038/s41467-025-56857-z (PMC11822037; doi:10.1038/s41467-025-56857-z)
Supplement: Supplementary file 1 — Supplementary Information [file 41467_2025_56857_MOESM1_ESM.pdf]

Supplementary materials

The systemic lupus erythematosus-associated NCF1^{90H} allele synergizes with viral infection to cause mouse lupus but also limit virus spread

Yanpeng Li[1, 2], Ana Coelho[1], Zhilei Li[3], Malin Alsved[4], Qixing Li[2], Rui Xu[2], Huqiao Luo[1], Dongxia Liang[5], Jing Xu[6], Kutty Selva Nandakumar[2], Liesu Meng[5, 6], Jakob Löndahl[4], Rikard Holmdahl[1, 2, 5]*

[1] Medical Inflammation Research, Division of Immunology, Department of Medical Biochemistry and Biophysics, Karolinska Institute, 17177 Stockholm, Sweden

[2] SMU-KI United Medical Inflammation Center, School of Pharmaceutical Sciences, Southern Medical University, 510515 Guangzhou, China

[3] Clinical Pharmacy Division, Department of Pharmacy, Southern University of Science and Technology Hospital, 518055 Shenzhen, China

[4] Division of Ergonomics and Aerosol Technology, Faculty of Engineering, Lund University, 22100 Lund, Sweden

[5] National and Local Joint Engineering Research Center of Biodiagnosis and Biotherapy, Second Affiliated Hospital of Xi' an Jiaotong University (Xibei Hospital), 710004 Xi' an, China.

[6] Key Laboratory of Environment and Genes Related to Diseases (Xi'an Jiaotong University), Ministry of Education, 710004 Xi'an, China.

***Corresponding author:** Rikard Holmdahl, email: rikard.holmdahl@ki.se

Table of contents

- 1. Supplementary figures
- 2. Supplementary tables
- 3. Supplementary Note 1

Supplementary Figures

Supplementary Fig. 1

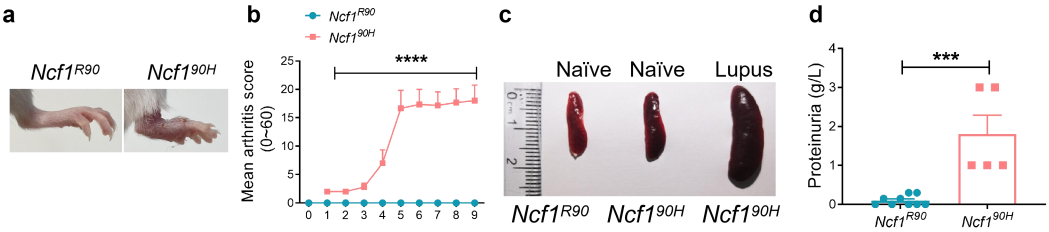

Supplementary Fig. 1 Spontaneous development of arthritis and lupus were noted in eight-week-old male BALB/c.*Ncf1^{90H}* mice. **a** Representative paws. **b** Mean arthritis score. **c** Representative spleen. Data were analyzed by Mann-Whitney test (two-tailed) and presented as mean± SEM. **d** Proteinuria in *Ncf1^{R90}* (n = 9) and *Ncf1^{90H}* mice (n = 5). Data were analyzed by Mann-Whitney test (two-tailed) and presented as mean±SEM. ***p < 0. 001; ****p < 0. 0001.

Supplementary Fig. 2

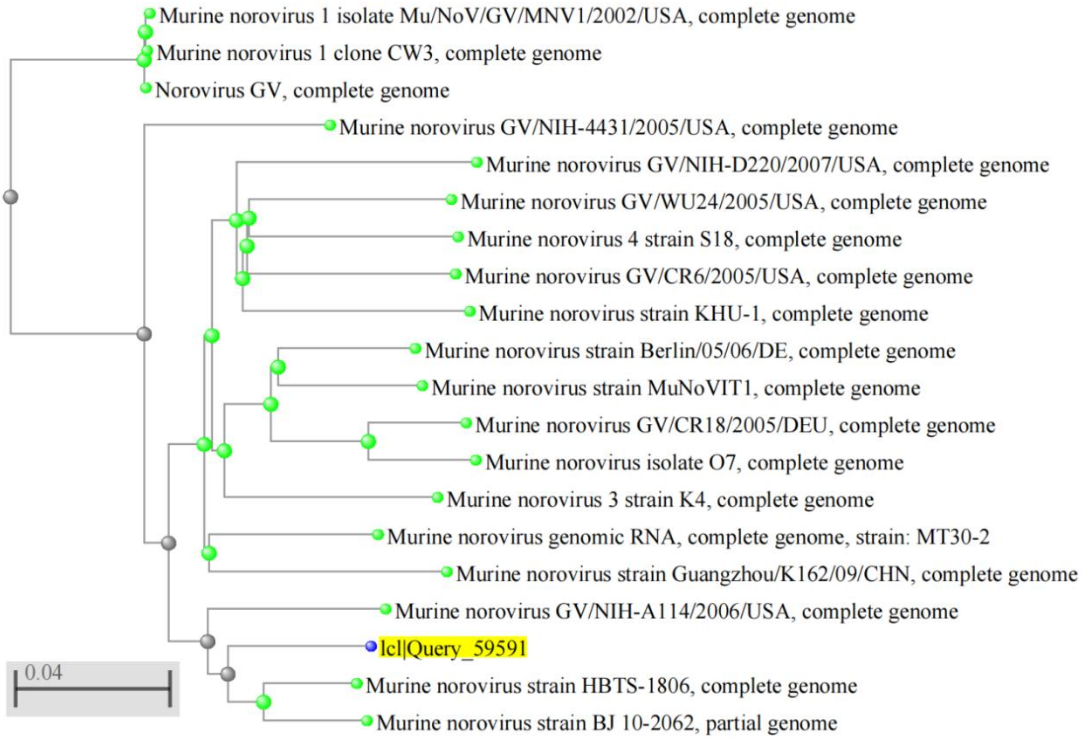

Supplementary Fig. 2 Phylogenetic relationships between MNV isolates with other known MNVs. Nucleotide sequences were compared for the whole genome. The scale in these phylogenetic trees represents that a given length along the branches of the tree corresponds to 0.04 substitutions per site in the genetic sequence.

Supplementary Fig. 3

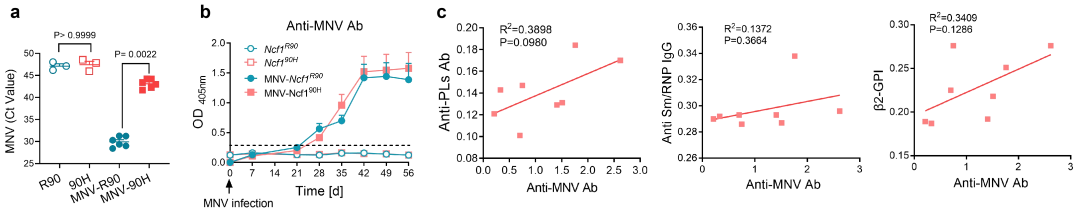

Supplementary Fig. 3 Environmental MNV induces lupus in male BALB/c. *Ncf1^{90H}* mice. a virus RNA was detected from the feces collected on day 7 after exposed into the environmental MNV. RT-qPCR was performed to assess MNV load with Ct value. Data were analyzed by Mann-Whitney test (two-tailed) and presented as mean ± SEM. **b** The levels of anti-MNV antibodies in mouse serum after infection with MNV from days 0 to 56, quantified by ELISA. **c** Correlation analysis between the levels of anti-MNV antibodies with anti-phospholipid (PLs), anti-Sm/RNP IgG antibodies and β2-GP1 antigens in BALB/c. *Ncf1^{90H}* mice on day 56 (n = 8). Data were analyzed using Pearson correlation test.

Supplementary Fig. 4

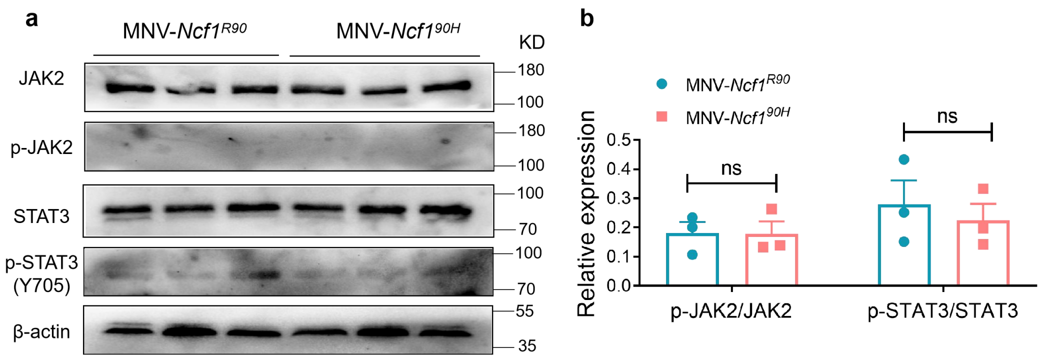

Supplementary Fig. 4 MNV-induced lupus. *Ncf1*^{90H} allele had no effect on JAK2-STAT3 pathway in the kidneys of MNV infected *Ncf1*^{90H} mice (n = 3). **a.** Immunoblot analysis of p-JAK2/JAK2 and p-STAT3/STAT3 proteins in the kidneys on day 56 after MNV infection (n = 3 per group). **b.** Statistics. Data were analyzed by Mann-Whitney test (two-tailed) and presented as mean±SEM.

Supplementary Fig. 5

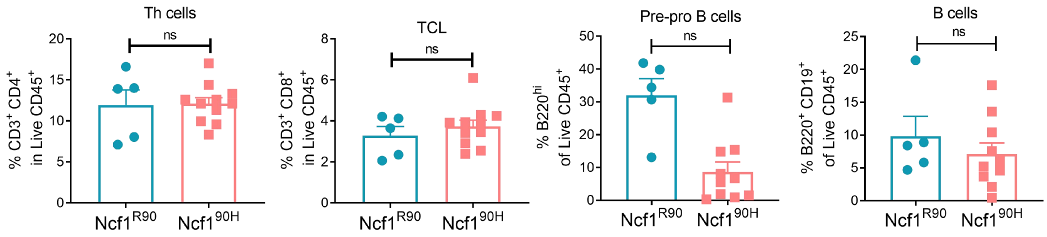

Supplementary Fig. 5 Pristane-induced lupus. The population of Th (CD45^{+} CD3^{+} CD4^{+}), TCL (CD45^{+} CD3^{+} CD8^{+}), Pre-pro B (CD45^{+} B220^{hi}), and B (CD45^{+} B220^{+} CD19^{+}) cells in the peritoneal cavity three days post-injection of pristane (R90: n = 5; 90H; n = 10) . Data were analyzed by Mann-Whitney test (two-tailed) and presented as mean±SEM.

Supplementary Fig. 6

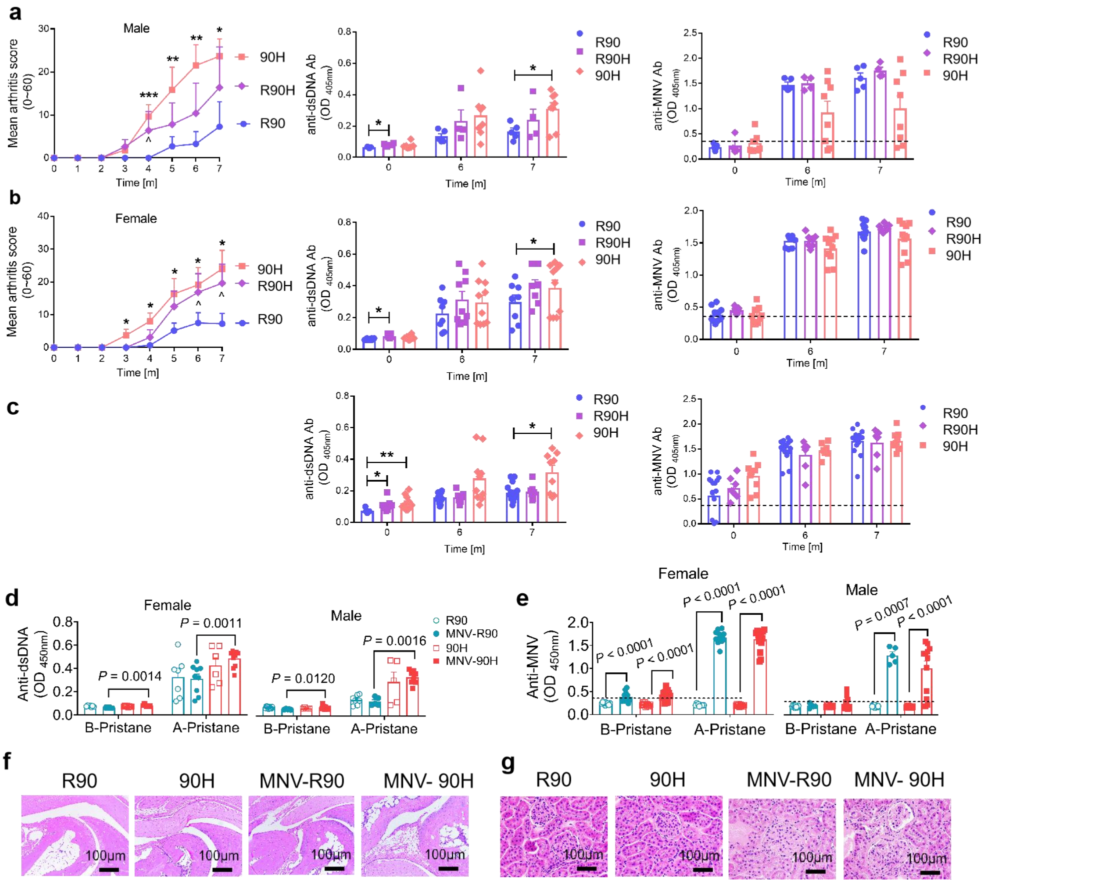

Supplementary Fig. 6 MNV aggravates pristane-induced lupus in BQ. ***Ncf1*^{90H} mice. a, b and c** Mean arthritis with BQ background in males (R90: n = 7; R90H: n = 5; 90H: n = 8) and females (R90: n = 12; R90H: n = 9; 90H: n = 12).Arthritis did not develop in the female B6 background mice. Anti-dsDNA antibodies with BQ background in males (R90: n = 5; R90H: n = 4; 90H: n = 8) and females (R90: n = 8; R90H: n = 9; 90H: n = 10) and with B6 background in females (R90: n = 14; R90H: n = 9; 90H: n = 11). Anti-MNV antibodieswith BQ background in males (R90: n = 5; R90H: n = 4; 90H: n = 8) and females (R90: n = 11; R90H: n = 7; 90H: n = 10), with B6 background in females (R90: n = 16; R90H: n = 7; 90H: n = 9). Data were analyzed by Mann-Whitney test (two-tailed) and presented as mean ± SEM. Data were analyzed by Mann-Whitney test (two-tailed) and presented as mean ± SEM. R90 vs 90H: * p < 0.05; R90 vs R90H: ^ p < 0.05. **d** Levels of anti-dsDNA antibodies before (B-Pristane) and after seven-month injection of pristane (A-Pristane) in females (R90H: n = 7; MNV-R90: n = 9; 90H: n = 8; MNV-90H: n = 10) and males (R90H: n = 10; MNV-R90: n = 5; 90H: n = 5; MNV-90H: n = 10). Data were analyzed by Mann-Whitney test (two-tailed) and presented as mean ± SEM. **e** Levels of anti-MNV antibodies before (B-Pristane) and after seven-month injection of pristane (A-Pristane) in females (R90H: n = 10; MNV-R90: n = 11; 90H: n = 10; MNV-90H: n = 17) and males (R90H: n = 10; MNV-R90: n = 5; 90H: n = 10; MNV-90H: n = 12). Data were analyzed by Mann-Whitney test (two-tailed) and presented as mean±SEM. **f, g** Representative HE staining of joints and kidneys after six months of pristane injections together with MNV infection (n = 4 per group).

Supplementary Fig. 7

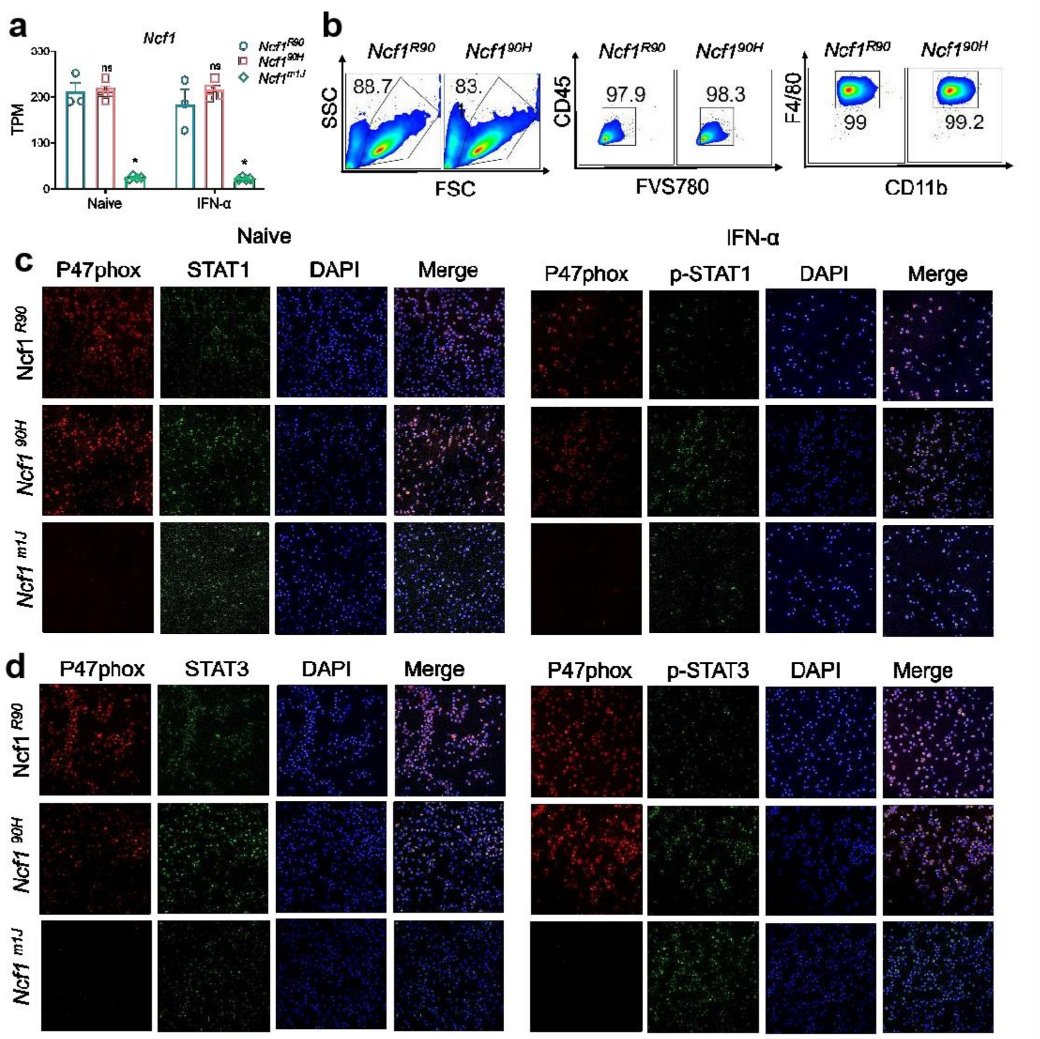

Supplementary Fig. 7 The role of *Ncf1*^{90H} allele. a *Ncf1*^{90H} allele had no effect on *Ncf1* mRNA expression. Data were analyzed by Mann-Whitney test (two-tailed) and presented as mean ± SEM. **b** The ratio of mature macrophages (CD11b^{+} F4/80^{+} % CD45^{+}) cultured with M-CSF is also shown (n = 4)**. c, d** Immunofluorescence staining of STAT1 and STAT3 in naïve *Ncf1*^{90H} macrophages and p-STAT1 and p-STAT3 in IFN-α stimulated *Ncf1*^{90H} macrophages expressing intact NCF1 (p47phox) (n = 4).

Supplementary Fig. 8

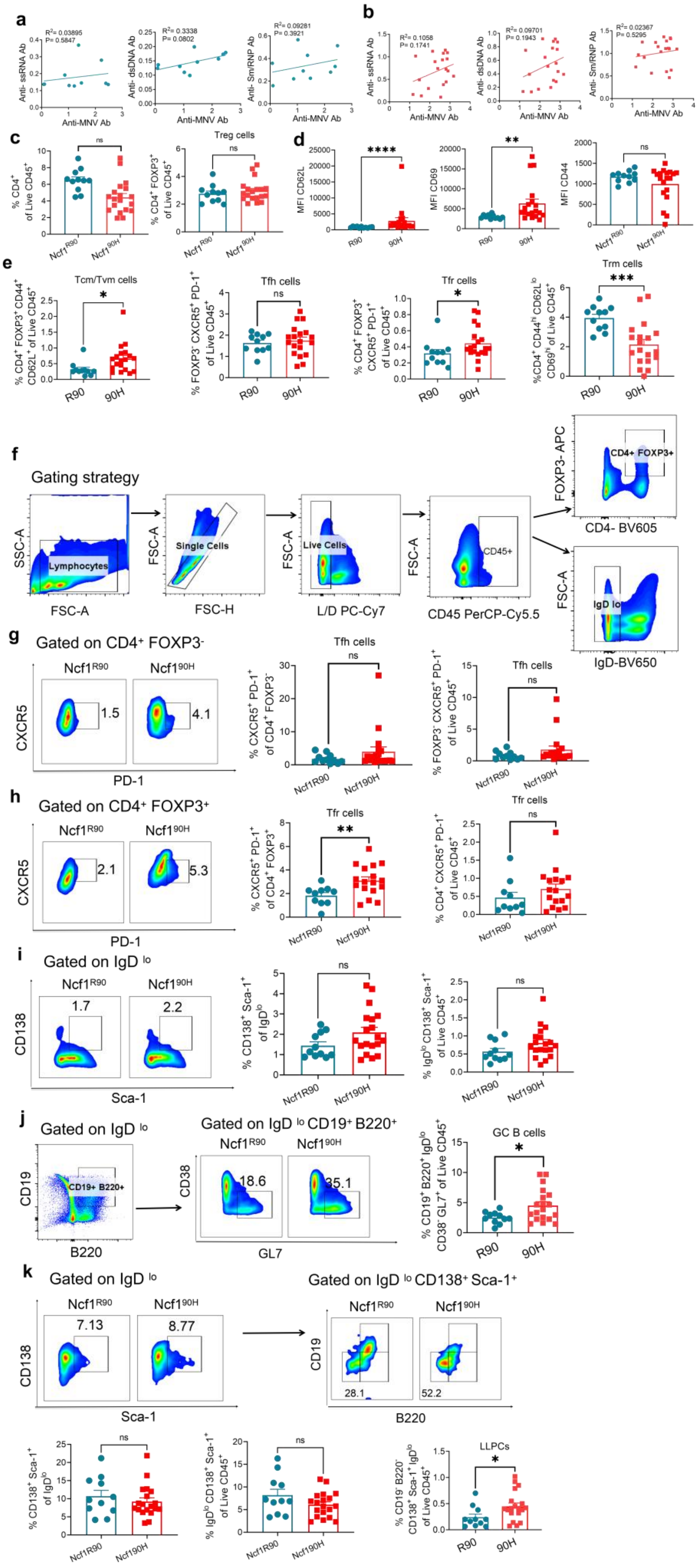

Supplementary Fig. 8 Mucosal-MNV induced lupus. a, b correlation between lupus associated autoantibodies against ssRNA, dsDNA, and Sm/RNP and anti-MNV antibodies in mouse sera of wildtype *Ncf1^R90^* (n = 11) and *Ncf1^90H^* mice (n = 12), respectively. Data were analyzed using Pearson correlation test. **c** The population of T helper cells (Th, $CD4^+$) or regulatory T cells (Tregs, $CD4^+$ $FOXP3^+$) cells in live $CD45^+$ cells from the Peyer's patches (PPs) (Ncf1R90: n = 11; Ncf190H: n = 19) . Data were analyzed by Mann-Whitney test (two-tailed) and presented as mean±SEM. **d** The expression of CD62L, CD69 and CD44 on $CD4^+$ T cell. **e** The ratio of Tcm or Tvm cells,Tfh cells, Tfr cells or Trm cells in Live $CD45^+$ in the PPs (Ncf1R90: n = 11; Ncf190H: n = 19). Data were analyzed by Mann-Whitney test (two-tailed) and presented as mean±SEM. **f** Gating strategies of $CD4^+$ $FOXP3^+$ and IgD^{lo}. **g, h** The ratio of Tfh ($CD4^+$ $FOXP3^-$ $CXCR5^+$ PD-1^+) and Tfr ($CD4^+$ $FOXP3^+$ $CXCR5^+$ PD-1^+) cells from the small intestine without PPs (Ncf1R90: n = 11; Ncf190H: n = 19). Data were analyzed by Mann-Whitney test (two-tailed) and presented as mean±SEM. **i** The population of antibody-secreting cells (ASCs) (IgD^{lo} $CD138^+$ Sca-1^+) in IgD^{lo} B cells from PPs and the small intestine without PPs (Ncf1R90: n = 11; Ncf190H: n = 19). Data were analyzed by Mann-Whitney test (two-tailed) and presented as mean±SEM. **j** The ratio of GC-B cells in Live $CD45^+$ cells in the PPs (Ncf1R90: n = 11; Ncf190H: n = 19) . Data were analyzed by Mann-Whitney test (two-tailed) and presented as mean±SEM. **k** The ratio of LLPCs in Live $CD45^+$ cells in the PPs (Ncf1R90: n = 11; Ncf190H: n = 19). Data were analyzed by Mann-Whitney test (two-tailed) and presented as mean±SEM. (n = 10-20).

Supplementary Fig. 9

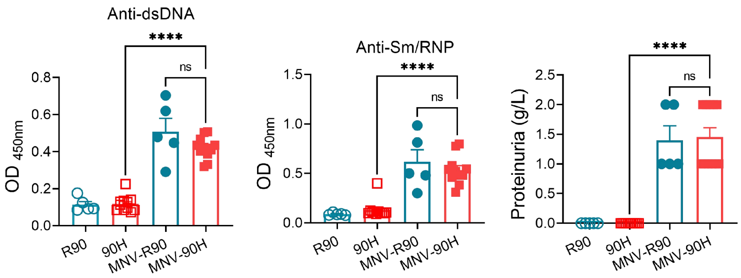

Supplementary Fig. 9 The level of anti-dsDNA, anti-Sm/ RNP and proteinuria on day 56 in non-mucosal MNV-induced lupus (R90: n = 6; 90H: n = 6; MNV-R90: n = 11; MNV-90H: n = 12). Data were analyzed using one-way ANOVA and presented as mean±SEM.

Supplementary Fig. 10

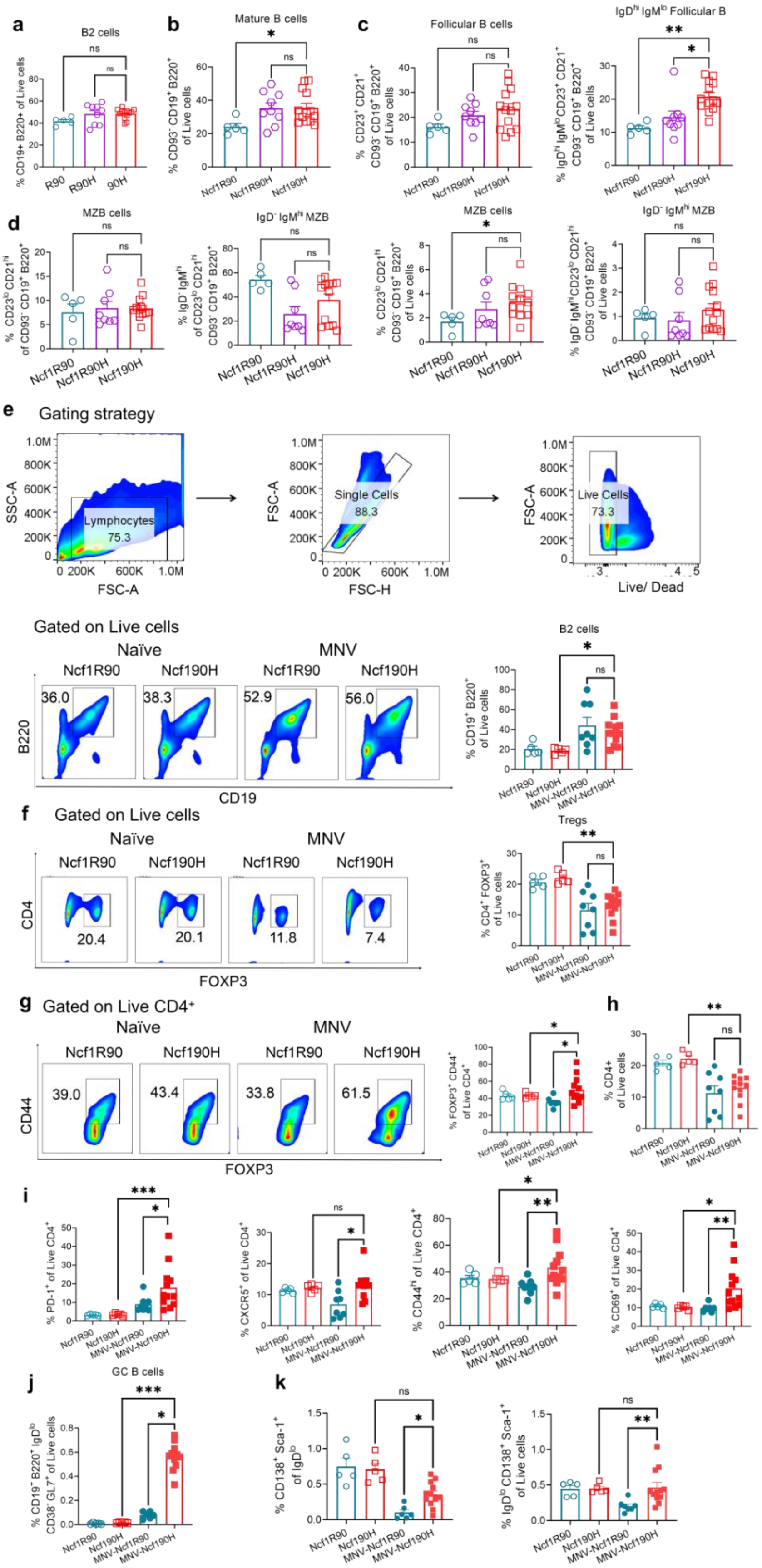

Supplementary Fig. 10 The maturation and differentiation of B and T cells. a The frequency of B2 cells ($CD19^{+}$ $B220^{+}$, gated on Live cells) (R90: n = 5; R90H: n = 9; 90H: n = 13). Data were analyzed using one-way ANOVA and presented as mean ± SEM. **b** The frequency of mature B ($CD93^{-}$, gated on $CD19^{+}$ $B220^{+}$) (R90: n = 5; R90H: n = 9; 90H: n = 13). Data were analyzed using one-way ANOVA and presented as mean ± SEM. **c** Follicular B ($CD23^{+}$ $CD21^{+}$, gated on $CD19^{+}$ $B220^{+}$ $CD93^{-}$), and IgD^{hi} IgM^{lo} Follicular B (IgD^{hi} IgM^{lo}, gated on $CD23^{+}$ $CD21^{+}$ $CD19^{+}$ $B220^{+}$ $CD93^{-}$) cells (R90: n = 5; R90H: n = 9; 90H: n = 13) . Data were analyzed using one-way ANOVA and presented as mean ± SEM. **d** MZB cells ($CD23^{lo}$ $CD21^{hi}$, gated on $CD93^{-}$ $CD19^{+}$ $B220^{+}$), IgD^{-} IgM^{hi} MZB (IgD^{-} IgM^{hi}, gated on $CD23^{lo}$ $CD21^{hi}$ $CD93^{-}$ $CD19^{+}$ $B220^{+}$), MZB and IgD^{-} IgM^{hi} MZB cells in Live cells (R90: n = 5; R90H: n = 9; 90H: n = 13). Data were analyzed using one-way ANOVA and presented as mean ± SEM. **e** Gating strategies of live cells. The population of B2 cells (R90: n = 5; 90H: n = 5; MNV-R90: n = 8; MNV-90H: n = 12). Data were analyzed using one-way ANOVA and presented as mean ± SEM. **f** The frequency of Tregs ($CD4^{+}$ $FOXP3^{+}$, gated on Live cells) (R90: n = 5; 90H: n = 5; MNV-R90: n = 8; MNV-90H: n = 12). Data were analyzed using one-way ANOVA and presented as mean±SEM. **g** The population of of $CD44^{+}$ Tregs cells (R90: n = 5; 90H: n = 5; MNV-R90: n = 8; MNV-90H: n = 12). Data were analyzed using one-way ANOVA and presented as mean ± SEM. **h** The population of Th cells ($CD4^{+}$, gated on Live cells) (R90: n = 5; 90H: n = 5; MNV-R90: n = 8; MNV-90H: n = 12). Data were analyzed using one-way ANOVA and presented as mean ± SEM. **i** The population of PD-1^{+}, $CXCR5^{+}$, $CD44^{+}$, and $CD69^{+}$ on Th cells (R90: n = 5; 90H: n = 5; MNV-R90: n = 8; MNV-90H: n = 12). Data were analyzed using one-way ANOVA and presented as mean ± SEM. **j** Germinal center B cells ($CD38^{lo}$ $GL7^{+}$ cells, gated on $B220^{+}$ $CD19^{+}$ IgD^{-} population)(R90: n = 5; 90H: n = 5; MNV-R90: n = 8; MNV-90H: n = 12) . Data were analyzed using one-way ANOVA and presented as mean±SEM. **k** The population of antibody-secreting cells (ASCs) (IgD^{lo} $CD138^{+}$ Sca-1^{+}) (R90: n = 5; 90H: n = 5; MNV-R90: n = 8; MNV-90H: n = 12). Data were analyzed using one-way ANOVA and presented as mean±SEM.

Supplementary Fig. 11

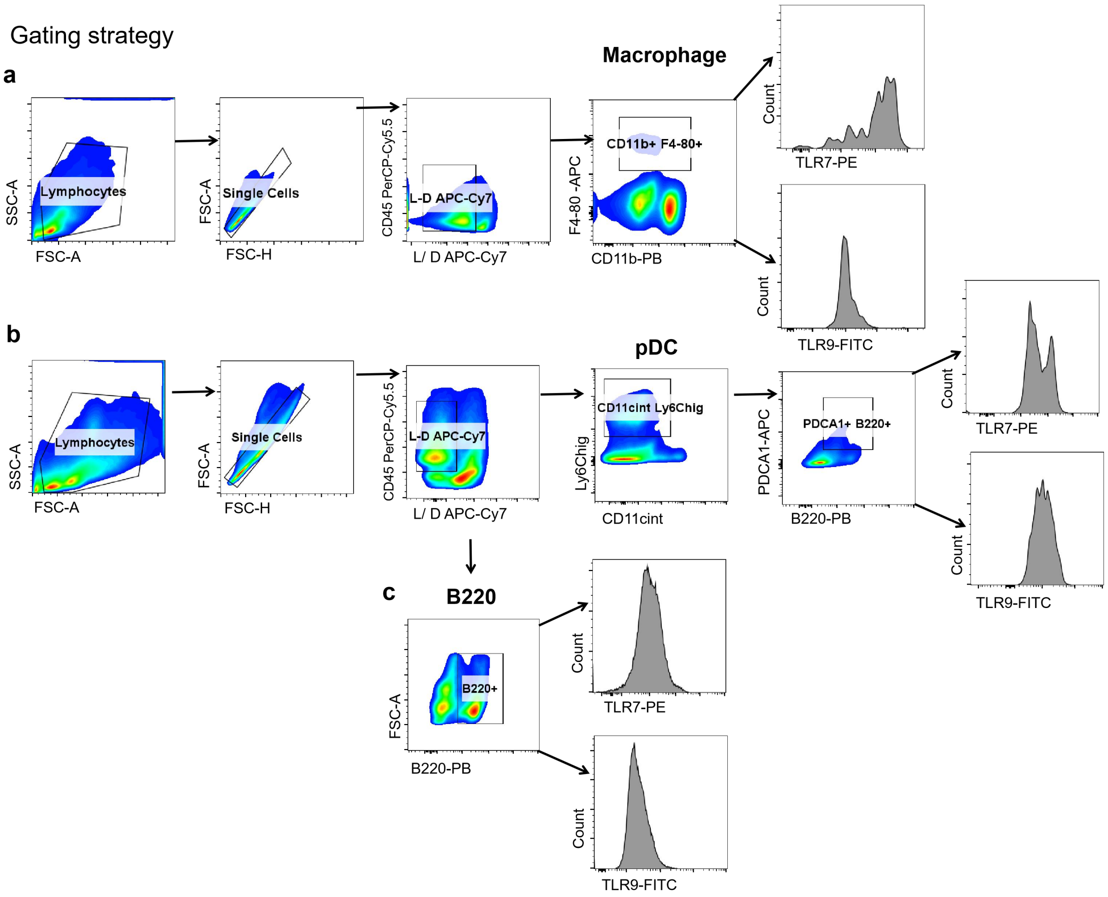

Supplementary Fig. 11 Gating strategy of TLR7 and TLR9 expression in immune cells. a macrophages (CD45^{+} CD11b^{+} F4/80^{+}). **b** pDCs (CD11c^{int} Ly6C^{hi} PDCA-1^{+} B220^{+}). **c** B cells (B220^{+}).

Supplementary Fig. 12

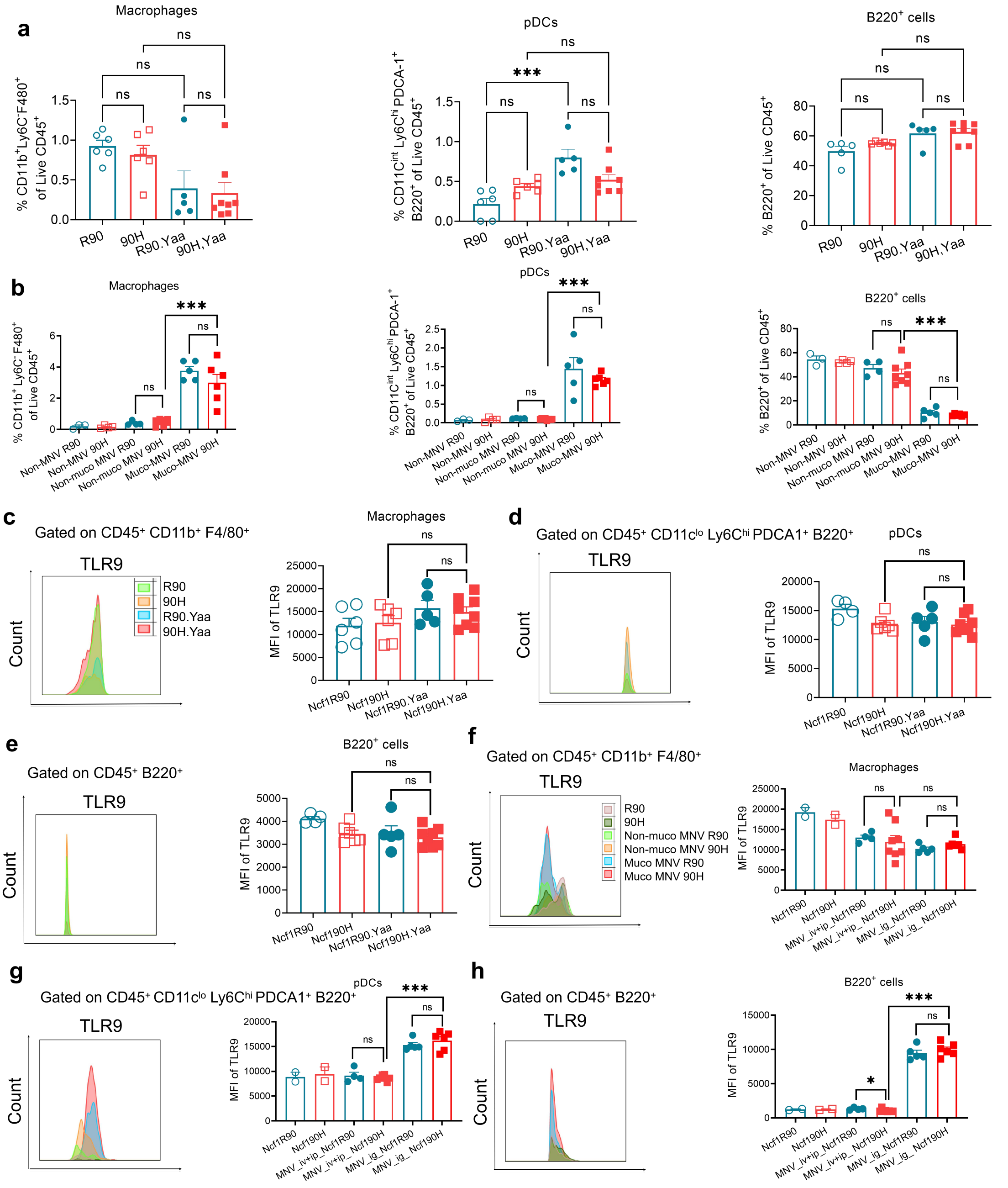

Supplementary Fig. 12 The effect of Yaa locus and MNV on TLR9 expression in Ncf1^{90H} mice. The Yaa-carrying mice strain with fully functional NCF1 (R90.Yaa) or with human NCF1^{90H} allele (90H.Yaa) was obtained by crossing homozygous BQ.*Ncf1*$^{90H/90H}$ with B10.Q.Yaa. And heterozygous BQ.*Ncf1*$^{R90/90H}$.Yaa were intercrossed for the experimental mice. **a** The population of macrophages, pDCs, and B220^{+} cells in naïve mice with and without *Yaa* locus (R90: n = 6; 90H: n = 6; R90.Yaa: n = 5; 90H.Yaa: n = 8). Data were analyzed using one-way ANOVA and presented as mean ± SEM. **b** The population of macrophages (Non MNV-R90: n = 2; Non MNV-90H: n = 2; Non-muco MNV-R90: n = 4; Non-muco MNV-90H: n = 8; Muco MNV-R90: n = 5; Muco MNV-90H: n = 6), pDCs (Non MNV-R90: n = 2; Non MNV-90H: n = 2; Non-muco MNV-R90: n = 4; Non-muco MNV-90H: n = 8; Muco MNV-R90: n = 5; Muco MNV-90H: n = 6), and B220^{+} cells (Non MNV-R90: n = 2; Non

MNV-90H: n = 2; Non-muco MNV-R90: n = 4; Non-muco MNV-90H: n = 8; Muco MNV-R90: n = 5; Muco MNV-90H: n = 6) in non-MNV infected mice, non-mucosal MNV infected and mucosal MNV infected mice. Data were analyzed using one-way ANOVA and presented as mean ± SEM. **c-e** Flow gating and analysis of TLR9 in naïve *Ncf1^{90H}* mice with *Yaa* locus in macrophages, pDCs, and B cells (R90: n = 6; 90H: n = 6; R90.Yaa: n = 5; 90H.Yaa: n = 8). Data were analyzed using one-way ANOVA and presented as mean±SEM. **f-h** Flow gating and analysis of TLR9 in *Ncf1^{90H}* mice with and without MNV infection, in macrophages, pDCs, and B cells (Non MNV-R90: n = 2; Non MNV-90H: n = 2; Non-muco MNV-R90: n = 4; Non-muco MNV-90H: n = 8; Muco MNV-R90: n = 5; Muco MNV-90H: n = 6). Data were analyzed using one-way ANOVA and presented as mean±SEM.

Supplementary Tables

Supplementary Table 1: Test results showing the presence of MNV and MNV Abs in mice.

		RESULTS			
TEST ITEM	TESTED	POS (+)	IND (±)	NEG (-)	PEN (?)
ECTV Ab	16	0	0	16	0
MHV Ab	16	0	0	16	0
SV Ab	16	0	0	16	0
Reo-3 Ab	16	0	0	16	0
PVM Ab	16	0	0	16	0
MVM Ab	16	0	0	16	0
SALM	16	0	0	16	0
CPIL Ab	16	0	0	16	0
MYCO Ab	16	0	0	16	0
CKUT	16	0	0	16	0
PPNE	16	0	0	16	0
KPNE	16	0	0	16	0
SAUR	16	0	0	16	0
PAER	16	0	0	16	0
ECTO	16	0	0	16	0
TOXO Ab	16	0	0	16	0
HELM	16	0	0	16	0
FLAG	16	0	0	16	0
CIL1	16	0	0	16	0
MNV	16	16	0	0	0
MNV Ab	16	16	0	0	0

* $Ncf1^{R90}$ (n = 8) and $Ncf1^{90H}$ (n = 8) mice were used in the tests. n, denotes number of mice. Presence of different viruses and antibodies were tested using a standard protocol.

Supplementary Table 2: Major MNV nucleotide sequences.

	Score	Expect	Identities	Gaps	Strand
	97.1 bits (52)	7e-17	52/52 (100%)	0/52(0%)	
	Plus/Plus				
Isolate-59591	CCCGCAGGAACGCTCAGCAGTCTTTGTGAATGAGGATGAGTGATGGCGCAGC				
CW3	CCCGCAGGAACGCTCAGCAGTCTTTGTGAATGAGGATGAGTGATGGCGCAGC				
MNVSH1603	CCCGCAGGAACGCTCAGCAGTCTTTGTGAATGAGGATGAGTGATGGCGCAGC				
HBTS-1806	CCCGCAGGAACGCTCAGCAGTCTTTGTGAATGAGGATGAGTGATGGCGCAGC				
BJ 10-2062	CCCGCAGGAACGCTCAGCAGTCTTTGTGAATGAGGATGAGTGATGGCGCAGC				

*The region is from bases 5027 to 5078.

Supplementary Table 3: Oligonucleotide primers used in this study.

Name	Sequence (5′-3′)	Genomic position*
1F	GTGAAATGAGGATGGCAACG	1-20
1282R	AGTTGGCACTCGTTCTTGAT	1263-1282
851F	AAACCTTCTGGCATCTGTGA	851-870
1985R	CAAGATGAAATTGATGTGGC	1966-1985
1790F	CATCATCATCACCACCAACC	1790-1809
3442R	ACCCAGGTGTTTCCTTTCTT	3423-3442
3229F	TTGTCGCTTCGGTCCTTGTT	3229-3248
4816R	TGGTGATTGGGTCCTTTGGT	4797-4816
4388F	CCCTTCGCTGCTGGATGTTG	4368-4387
5967R	CACCTGACCCGTGCCTGATT	5948-5967
5454F	AGGGTCACTCACCACTGCTC	5454-5673
7382R	AAAATGCATCTAATTACTAC	7363-7382R

*Location corresponds to position within the BJ 10-2062 (KM458057) genome.

Supplementary Table 4: RT-qPCR primer sequences for mouse genes.

Gene	Forward (5'–3')	Reverse (5'–3')
Ncf1	GTGGTCTACAGAAAATTCACCGA	CCATGAGGCCGTTGAAGTATTC
Ncf2	TCACCAAGACAACTTTTCTGGC	GGCCCAGTTATCACTGCCC
Ncf4	GTCATCGAGGTCAAAACAAAAGG	GCCCATGTAGACTTTGGCTG
Mx1	GATCCGACTTCACTTCCAGATGG	CATCTCAGTGGTAGTCAACCC
Stat1	GGGCCCTAATGCTGGCCCTG	GCGCGTTCTCGCTCCTTGCT
Irf1	GCACCACTGATCTGTATAACCTACA	CCTCATCCTCGTCTGTTGC
Irf7	ACAGCACAGGGCGTTTTATC	GGGCCCACAGTAGATCCAAG
Ip10	GACGGTCCGCTGCAACTG	GCTTCCCTATGGCCCTCATT
Isg15	GAGCTAGAGCCTGCAGCAAT	TAAGACCGTCCTGGAGCACT
Ifnα	CGGACGCTGGATTAACACCT	TGCCAAGGTGGCTGTAGATG
β-actin	GGCTGTATTCCCCTCCATCG	CCAGTTGGTAACAATGCCATGT

Supplementary Note 1

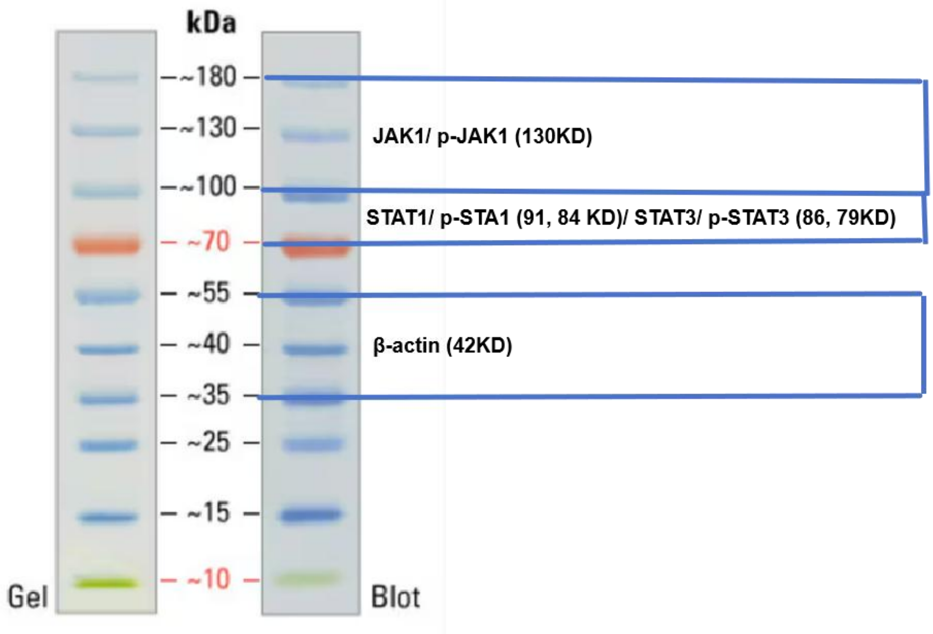

Explanation of methods for Western blots shown in figures 1, 3 and S4.

The gels were run with the indicated samples and transferred to blots (PVDF membrane) whereafter stripes of the blots were cut out based on the sizes as given by the weight standard of known proteins as given by the manufacturer (see figure). Thus, stripes presumably including p-JAK1 were cut between 100 and 180 kD, stripes presumably including p-STAT1 between 70-100 kD and stripes presumably including β-actin between 35-55 kD. These stripes were incubated in tubes together with the specific primary antibodies followed by incubation with secondary antibodies, whereafter they were developed with HRP and photographed. The stripes were reused after stripping of the used antibodies and stained again with a new set of primary antibodies.

Primary antibodies: Rabbit antibodies specific for JAK1 (CST, 3332), p-JAK-1 (CST, 3331) (100-180 kd stripes), rabbit antibodies specific for p-STAT1^{Tyr701} (CST, clone: 58D6, 88845), STAT1 (CST, clone: D1K9Y, 65748), P-STAT3^{Tyr705} (CST, clone: Tyr705, 9131), or STAT3 (CST, clone: 79D7, 4904) (70-100 kD), and rabbit antibodies specific for β-actin (CST, 4967) (35-55 kD).

Secondary antibodies: anti-rabbit IgG conjugated with HRP (1:4,000 dilutions; Southern Biotech, 4030-05).

Primary antibody incubations were done in tubes with 15ml diluent at 4℃ overnight and washed with TBST between steps.

Secondary antibody incubations were done in tubes with 15 ml PBS for one hour in room temperature and washed with TBST between steps.

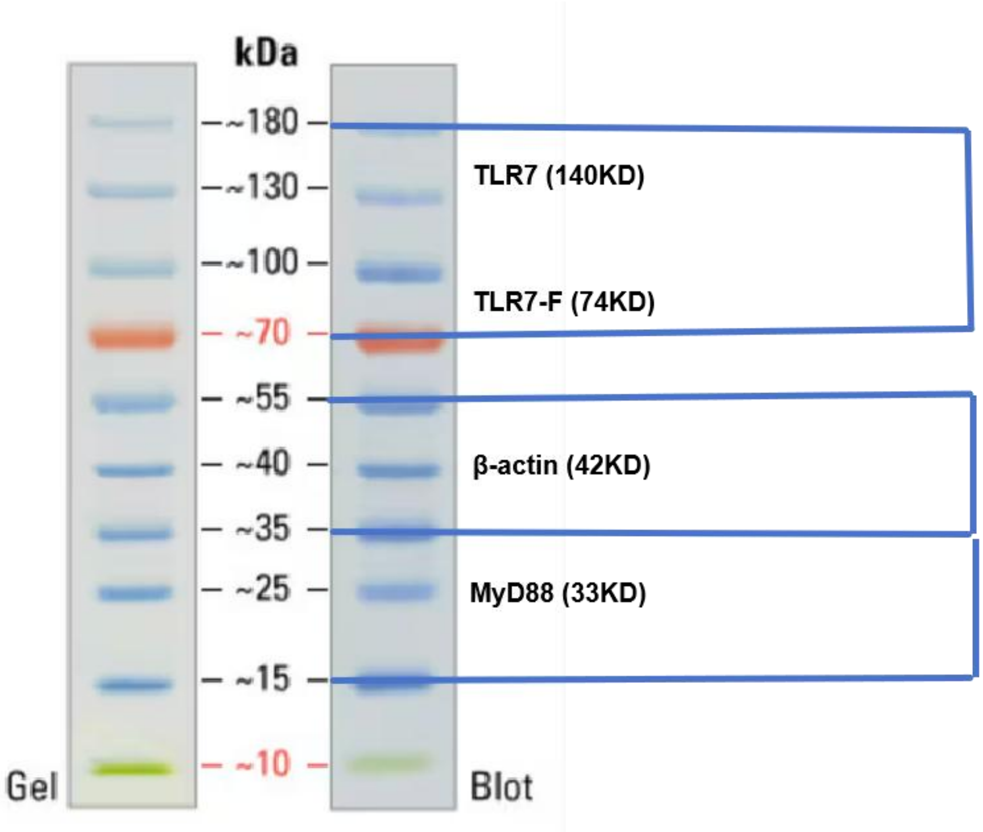

Explanation of methods for Western blots shown in figure 7.

The gels were run with the indicated samples and transferred to blots (PVDF membrane) whereafter stripes of the blots were cut out based on the sizes as given by the weight standard of known proteins as given by the manufacturer (see figure). Thus, stripes presumably including TLR7/TLR7-F were cut between 70 and 180 kD, stripes presumably including b-actin between 35-55 kD and stripes presumably including myd88 between 15-35 kD. These stripes were incubated in 15 ML tubes together with the specific primary antibodies followed by incubation with secondary antibodies, whereafter they were developed with HRP and photographed together with the standard weight blots.

Primary antibodies: Rabbit antibodies specific for TLR7 or TLR7-F (CST, D7, 5632) (70-180 KD stripes). Rabbit antibodies specific for β-actin (CST, 4967) (35-55 kD stripes). Rabbit antibodies specific for MyD88 (CST, D80F5, 4283) (15-35 kD stripes).

Secondary antibodies: anti-rabbit IgG conjugated with HRP (1:4,000 dilutions; Southern Biotech, 4030-05).

Primary antibody incubations were done in tubes with 15ml diluent at 4℃ overnight and washed with TBST between steps.

Secondary antibody incubations were done in tubes with 15 ml PBS for one hour in room temperature and washed with TBST between steps.
