# Supplementary material for: The systemic lupus erythematosus-associated NCF190H allele synergizes with viral infection to cause mouse lupus but also limits virus spread
Source: Nat Commun. 2025 Feb 13;16:1593. doi: 10.1038/s41467-025-56857-z (PMC11822037; doi:10.1038/s41467-025-56857-z)
Supplement: Supplementary file 2 — Reporting Summary [file 41467_2025_56857_MOESM2_ESM.pdf]

nature portfolio

Corresponding author(s): Rikard Holmdahl

Last updated by author(s): Jan 24, 2025

Reporting Summary

Nature Portfolio wishes to improve the reproducibility of the work that we publish. This form provides structure for consistency and transparency in reporting. For further information on Nature Portfolio policies, see our Editorial Policies and the Editorial Policy Checklist.

Statistics

For all statistical analyses, confirm that the following items are present in the figure legend, table legend, main text, or Methods section.

n/a	Confirmed	
	☒	The exact sample size (*n*) for each experimental group/condition, given as a discrete number and unit of measurement
	☒	A statement on whether measurements were taken from distinct samples or whether the same sample was measured repeatedly
	☒	The statistical test(s) used AND whether they are one- or two-sided<br>*Only common tests should be described solely by name; describe more complex techniques in the Methods section.*
☒		A description of all covariates tested
	☒	A description of any assumptions or corrections, such as tests of normality and adjustment for multiple comparisons
	☒	A full description of the statistical parameters including central tendency (e.g. means) or other basic estimates (e.g. regression coefficient) AND variation (e.g. standard deviation) or associated estimates of uncertainty (e.g. confidence intervals)
	☒	For null hypothesis testing, the test statistic (e.g. *F*, *t*, *r*) with confidence intervals, effect sizes, degrees of freedom and *P* value noted<br>*Give P values as exact values whenever suitable.*
☒		For Bayesian analysis, information on the choice of priors and Markov chain Monte Carlo settings
☒		For hierarchical and complex designs, identification of the appropriate level for tests and full reporting of outcomes
☒		Estimates of effect sizes (e.g. Cohen's *d*, Pearson's *r*), indicating how they were calculated

Our web collection on statistics for biologists contains articles on many of the points above.

Software and code

Policy information about availability of computer code

Data collection	BioTek Gen5 v1.05, Attune v5.2.0, LSR Fortessa(BD Biosciences), Bio-Rad CFX Manager v3.1, Zen Blue v3.1, Agilent Strata gene Mx3005P, LSM880 with Airyscan, BioSpot Software.
Data analysis	GraphPad Prism Software Version 9.5.0, Microsoft Office 16, FlowJo version 10.6, Image J.

For manuscripts utilizing custom algorithms or software that are central to the research but not yet described in published literature, software must be made available to editors and reviewers. We strongly encourage code deposition in a community repository (e.g. GitHub). See the Nature Portfolio guidelines for submitting code & software for further information.

Data

Policy information about availability of data

All manuscripts must include a data availability statement. This statement should provide the following information, where applicable:

- Accession codes, unique identifiers, or web links for publicly available datasets
- A description of any restrictions on data availability
- For clinical datasets or third party data, please ensure that the statement adheres to our policy

The data supporting the findings of this study are available within the paper (Supplementary Information). The other datasets used and/or analyzed during the current study are available from the corresponding author upon request. Source data are provided with this paper.

Research involving human participants, their data, or biological material

Policy information about studies with human participants or human data. See also policy information about sex, gender (identity/presentation), and sexual orientation and race, ethnicity and racism.

Reporting on sex and gender	N/A
Reporting on race, ethnicity, or other socially relevant groupings	N/A
Population characteristics	N/A
Recruitment	N/A
Ethics oversight	N/A

Note that full information on the approval of the study protocol must also be provided in the manuscript.

Field-specific reporting

Please select the one below that is the best fit for your research. If you are not sure, read the appropriate sections before making your selection.

[x] Life sciences [ ] Behavioural & social sciences [ ] Ecological, evolutionary & environmental sciences

For a reference copy of the document with all sections, see nature.com/documents/nr-reporting-summary-flat.pdf

Life sciences study design

All studies must disclose on these points even when the disclosure is negative.

Sample size	In environmental MNV infection experiment with BALB/c.Ncf190H mice, 8 male mice were included in each group. The male mice ((R90: n = 5; 90H; n = 10) were used in pristane-induced lupus model for population analysis of immune cells in the peritoneal cavity of 3 days after pristane injection. The female (R90: n = 9; 90H: n = 7; MNV-R90: n = 12; MNV-90H: n = 8) and male (R90: n = 7; 90H: n = 5; MNV-R90: n = 7; MNV-90H: n = 6) mice with and without MNV infection. MNV-R90 vs MNV-90H, non-MNV infected female mice (Pristane-R90: n = 7 and Pristane-90H: n = 6) and male mice (Pristane-R90: n = 7 and Pristane-90H: n = 5) and MNV infected female mice (MNV-Pristane-R90: n = 9 and MNV-Pristane-90H: n = 10) and male mice (MNV-Pristane-R90: n = 5 and MNV-Pristane-90H: n = 8) were used PIL model together with MNV infection. Murine BMDMs were obtained from the differentiation of monocytes recovered from the femur and tibia of 6 to 8- week- old male B6N.Q.Ncf1R90, B6N.Q. Ncf190H, B6N.Q and B6N.Q.Ncf1m1J mice (n = 5 per group). In MNV infection model by gavage, females (MNV-R90: n= 6; MNV-90H: n= 13) and males (MNV-R90: n = 5; MNV-90H: n = 6) were used. In MNV infection model by IV+IP injection, male (R90: n= 6; 90H: n = 6; MNV-R90: n= 11; MNV-90H: n = 12) mice were used. For the detection of STAT1 and p-STAT1 in peritoneal exudates macrophages one day post-MNV intraperitoneal injection, MNV-R90 (female: n = 4, male: n= 4) and MNV-90H (female: n= 5, male: n= 4) mice were used.
Data exclusions	No exclusion
Replication	Key experiments were replicated twice using identical protocols.
Randomization	Animals were randomly allocated to different groups, with littermates control strategy applied.
Blinding	All scoring procedures were carried out blindly for animal experiments. in other experiments, blinding strategy was applied when subjectiveevaluation was required, e.g., confocal imaging.

Reporting for specific materials, systems and methods

We require information from authors about some types of materials, experimental systems and methods used in many studies. Here, indicate whether each material, system or method listed is relevant to your study. If you are not sure if a list item applies to your research, read the appropriate section before selecting a response.

Materials & experimental systems

n/a	Involved in the study
	☒ Antibodies
	☒ Eukaryotic cell lines
☒	Palaeontology and archaeology
	☒ Animals and other organisms
☒	Clinical data
☒	Dual use research of concern
☒	Plants

Methods

n/a	Involved in the study
☒	ChIP-seq
	☒ Flow cytometry
☒	MRI-based neuroimaging

Antibodies

Antibodies used	Alexa Fluor 488-conjugated anti-mouse complement component C3 (1:200 dilutions; Cedarlane, CL7503AF4), Alexa Fluor 488-conjugated goat anti–mouse IgG specific for Fcγ fragment (1:500 dilutions, Jackson ImmunoResearch, 115-545-071), HRP-conjugated goat anti-mouse IgG (H+L) (1:4,000 dilutions; Southern Biotech, 1031-05), human ads-UNLB (SouthernBiotech, 1030-01), HRP-conjugated goat anti-mouse-IgG, -IgG2b, and -IgM (1:4,000 dilutions; Southern Biotech, 1030-05, 1090-05, 1020-05), human ads-BIOT (1:4000 dilutions; Southern Biotech, 1030-08), Streptavidin-HRP (1:800 dilutions, Southern Biotech, 7105-05), biotinylated goat anti-mouse IgG (1:1000 dilutions, Southern Biotech, 1030-08) or IgG2b (1:1000 dilutions, Southern Biotech, 1090-08), purified rat anti-mouse Fc-block (CD16/CD32, 24G2, homemade, BD Biosciences), CD45-HV500, Ly6C-BV605, Ly6G-APC, F4/80-PE, B220-Fluor50, CD19-PE, CD3-APC, CD4-PerCP/Cyanine5.5 and CD8a-FITC antibodies (Biolegend), CD19- PE- Cy7, B220-PB, CD93-PE, CD21-APC, CD23-PerCP-Cy5.5, IgM-BV605, IgD-BV650, B220-APC, MHCII-FITC, CXCR4-PerCP-Cy5.5, CXCR5-BV421, CD4-BV605, CD44-AF700, CD62L-FITC, CXCR5-BV421, PD-1-PE-Cy7, FOXP3-APC, CD93-PE, CD19-AF700, CD138-BV605, GL7-APC, CD38-PE, Sca-1-PE-Cy7, CD69-APC, CXCR5-PB, PD-1-PE, CCR6-PE- Cy7, CXCR3 (CD183)-PerCP-Cy5.5, CD69-PE antibodies (Biolegend), CD45-HV500, CD11b-PE-Cy7, Ly6C-BV605, Ly6G-APC, F4/80-FITC (Biolegend), PE Mouse anti-Total Stat1 (1:50 dilutions; BD Phosflow, N-Terminus, 558537) and BV421 Mouse anti-p-STAT1Tyr701 antibody (1:50 dilutions; BD Phosflow, 566238), CD45-PerCP-Cy5.5, CD11b-PB, F4/80-APC, Ly6C-BV605, CD11c-PE-Cy7, CD19-AF700, B220-PB, PDCA1 (CD317)-APC antibodies (Biolegend), TLR7-PE (BioLegend, clone: A94B10, 160003), and TLR9-FITC (BioLegend, clone: S18025A, 159107) antibodies, fixable near-IR dead cell stain kit (Thermo Fisher Scientific, L10119), mouse anti-p47phox (D-10) antibodies (Santa Cruz Biotechnology, sc-17845), or STAT1 (CST, clone: D1K9Y, 65748), p-STAT1Tyr701 (CST, clone: 58D6, 88845), STAT3 (CST, clone: 79D7, 4904), and p-STAT3Tyr705 (CST, clone: Tyr705, 9131) specific rabbit antibodies (1:1000 dilutions), goat anti-mouse IgG (H+L) (1:1000 dilutions; CST, 4408) or goat anti-rabbit IgG (H+L)-Alexa Fluor 488 (1:1000 dilutions; CST, 4412), mouse ani-NCF1 (p47phox) (D-10) antibodies (Santa Cruz Biotechnology, sc-17845) or STAT1 (CST, clone: D1K9Y, 65748), p-STAT1Tyr701 (CST, clone: 58D6, 88845), STAT3 (CST, clone: 79D7, 4904), and p-STAT3Tyr705 (CST, clone: Tyr705, 9131) specific rabbit antibodies (1:1000 dilutions), TLR-7 (CST, D7, 5632), MyD88 (CST, D80F5, 4283) and β-Actin (CST, 4967) specific rabbit antibodies, goat anti-mouse IgG conjugated with HRP (1:4,000 dilutions; Southern Biotech, 1031-05), anti-rabbit IgG conjugated with HRP (1:4,000 dilutions; Southern Biotech, 4030-05).
Validation	The commercial antibodies used for flow-cytometry, western blot,ELISA, ELISpot, immunofluorescent stalning et al., were validatedby the successful separation of the given positive and negative populations, aswell as by referring to the the validation statementsfrom manufactures.

Eukaryotic cell lines

Policy information about cell lines and Sex and Gender in Research

Cell line source(s)	RAW 264.7 (ATCC TIB-71, RRID:CVCL_0493), established from an ascites of a tumour induced in a male mouse by intraperitoneal injection of Abelson Leukaemia Virus (A-MuLV). Murine bone marrow derived macrophages (BMDMs) were obtained from the differentiation of monocytes recovered from the femur and tibia of 6 to 8- week-old male B6N.Q.Ncf1R90, B6N.Q. Ncf190H, B6N.Q and B6N.Q.Ncf1m1J mice.
Authentication	RAW 264.7 was analyzed by ATCC's FTA Sample Collection Kit for Mouse Cell Authentication Service, which uses short tandem repeat (STR) profiling.
Mycoplasma contamination	All cells were tested free of Mycoplasma contamination.
Commonly misidentified lines (See ICLAC register)	No commonly misidentified lines were used.

Animals and other research organisms

Policy information about studies involving animals; ARRIVE guidelines recommended for reporting animal research, and Sex and Gender in Research

Laboratory animals	Mouse strains used in this study: Balb/cByJ, C57BL/6NJ,C57/B6N.Q/rhd, C57/B10N.Q/rhd,DBA/1,BQ.Ncf190H,BQ.Ncf1m1J,BALB/c.Ncf190H,B10.Q,B10.Q.Yaa,B10.Q.Ncf1R90.Yaa,10.Q.Ncf190H.Yaa.Age-and sex-matched littermates from heterozygousintercrosses were used in all experiments. The facilities have a climate-controlled environment with a 14 h light/10 hrs dark cycle.The animals were housed in individually ventilated polystyrene cages containing enrichments with standard chow and water given adlibitum. The protocols were approved by local animal welfare authorities. For all the experiments, 4- to 8-week-old age-and sex-matched mutated mice and wild-type littermate controls were used.

Wild animals	No wild animals were used.
Reporting on sex	In environmental MNV infection experiment with BALB/c.Ncf190H mice, 8 male mice were included in each group. The male mice ((R90: n = 5; 90H; n = 10) were used in pristane-induced lupus model for population analysis of immune cells in the peritoneal cavity of 3 days after pristane injection. The female (R90: n = 9; 90H: n = 7; MNV-R90: n = 12; MNV-90H: n = 8) and male (R90: n = 7; 90H: n = 5; MNV-R90: n = 7; MNV-90H: n = 6) mice with and without MNV infection. MNV-R90 vs MNV-90H, non-MNV infected female mice (Pristane-R90: n = 7 and Pristane-90H: n = 6) and male mice (Pristane-R90: n = 7 and Pristane-90H: n = 5) and MNV infected female mice (MNV-Pristane-R90: n = 9 and MNV-Pristane-90H: n = 10) and male mice (MNV-Pristane-R90: n = 5 and MNV-Pristane-90H: n = 8) were used PIL model together with MNV infection. Murine BMDMs were obtained from the differentiation of monocytes recovered from the femur and tibia of 6 to 8- week- old male B6N.Q.Ncf1R90, B6N.Q. Ncf190H, B6N.Q and B6N.Q.Ncf1m1J mice (n = 5 per group). In MNV infection model by gavage, females (MNV-R90: n= 6; MNV-90H: n= 13) and males (MNV-R90: n = 5; MNV-90H: n = 6) were used. In MNV infection model by IV+IP injection, male (R90: n= 6; 90H: n = 6; MNV-R90: n= 11; MNV-90H: n = 12) mice were used. For the detection of STAT1 and p-STAT1 in peritoneal exudates macrophages one day post-MNV intraperitoneal injection, MNV-R90 (female: n = 4, male: n= 4) and MNV-90H (female: n= 5, male: n= 4) mice were used.
Field-collected samples	No field-collected samples were used.
Ethics oversight	All the experimental procedures were approved by the local ethical committee (Guangzhou, China, permit number: L2020013 orStockholm, Sweden, permit number: Dnr23517-2022,10523-2022and 2660-2019).All animal experiments were performedaccording to the ARRIVE guidelines.

Note that full information on the approval of the study protocol must also be provided in the manuscript.

Plants

Seed stocks	*Report on the source of all seed stocks or other plant material used. If applicable, state the seed stock centre and catalogue number. If plant specimens were collected from the field, describe the collection location, date and sampling procedures.*
Novel plant genotypes	*Describe the methods by which all novel plant genotypes were produced. This includes those generated by transgenic approaches, gene editing, chemical/radiation-based mutagenesis and hybridization. For transgenic lines, describe the transformation method, the number of independent lines analyzed and the generation upon which experiments were performed. For gene-edited lines, describe the editor used, the endogenous sequence targeted for editing, the targeting guide RNA sequence (if applicable) and how the editor was applied.*
Authentication	*Describe any authentication procedures for each seed stock used or novel genotype generated. Describe any experiments used to assess the effect of a mutation and, where applicable, how potential secondary effects (e.g. second site T-DNA insertions, mosiacism, off-target gene editing) were examined.*

Flow Cytometry

Plots

Confirm that:

- [x] The axis labels state the marker and fluorochrome used (e.g. CD4-FITC).
- [x] The axis scales are clearly visible. Include numbers along axes only for bottom left plot of group (a 'group' is an analysis of identical markers).
- [x] All plots are contour plots with outliers or pseudocolor plots.
- [x] A numerical value for number of cells or percentage (with statistics) is provided.

Methodology

Sample preparation	Briefly, organs were collected, mashed, and filtered through 45 μM filter to obtain single-cell suspensions in PBS. To prepare single-cell suspensions, the perfused kidneys were digested with 1 mg/mL collagenase (Roche, 11088866001) and 0.1 mg/mL DNase I (Roche, 10104159001) in a 37°C water bath for 45 minutes. Red blood cells were lysed using ammonium-chloride-potassium (ACK) buffer (homemade), cells were counted on a Sysmex, and purified rat anti-mouse Fc-block (CD16/CD32, 24G2, homemade, BD Biosciences; ≤ 1 μg/million cells in 100 μl) was added for 10 minutes at RT. Surface antigens were stained with fluorescently labeled antibodies. All the antibodies were used with 0.2 μg per million cells in 100 μl volume. To stain immune cells in the peritoneal cavity, spleens, and kidneys, CD45-HV500, Ly6C-BV605, Ly6G-APC, F4/80-PE, B220-Fluor50, CD19-PE, CD3-APC, CD4-PerCP/Cyanine5.5 and CD8a-FITC antibodies (Biolegend) were used. For the staining B and T cells, CD19-PE-Cy7, B220-PB, CD93-PE, CD21-APC, CD23-PerCP-Cy5.5, IgM-BV605, IgD-BV650, B220-APC, MHCII-FITC, CXCR4-PerCP-Cy5.5, CXCR5-BV421, CD4-BV605, CD44-AF700, CD62L-FITC, CXCR5-BV421, PD-1-PE-Cy7, FOXP3-APC, CD93-PE, CD19-AF700, CD138-BV605, GL7-APC, CD38-PE, Sca-1-PE-Cy7, CD69-APC, CXCR5-PB, PD-1-PE, CCR6-PE-Cy7, CXCR3 (CD183)-PerCP-Cy5.5, CD69-PE antibodies (Biolegend) were used. For detection of STAT1/p-STAT, peritoneal exudates cells were collected one day after MNV intraperitoneal injection at the dose of 3 x 105 TCID50, stained with CD45-HV500, CD11b-PE-Cy7, Ly6C-BV605, Ly6G-APC, F4/80-FITC (Biolegend), washed and fixed with Cytofix buffer (BD Biosciences) for 10 minutes at 37 °C, permeabilized with Phosflow Perm Buffer III on ice for 30 minutes, washed twice and then stained with PE Mouse anti-Total Stat1 (1:50 dilutions; BD Phosflow, N-Terminus, 558537) and BV421 Mouse anti-p-STAT1Tyr701 antibody (1:50 dilutions; BD Phosflow, 566238). For the detection of TLR7 and 9, splenocytes were collected, CD45-PerCP-Cy5.5, CD11b-PB, F4/80-APC, Ly6C-BV605, CD11c-PE-Cy7, CD19-AF700, B220-PB, PDCA1 (CD317)-APC antibodies (Biolegend) were used. Cells were fixed, permeabilized, and stained with TLR7-PE (BioLegend, clone: A94B10, 160003), and TLR9-FITC (BioLegend, clone: S18025A, 159107) antibodies. Dead cells were excluded using FVS780 (BD Biosciences, 565388) or a fixable near-IR dead cell stain kit

(Thermo Fisher Scientific, L10119).

Instrument	Attune v5.2.0 flow cytometer (Thermo Fisher) or using LSR Fortessa (BD Biosciences)
Software	FlowJo version 10.6
Cell population abundance	No sorting carried out.
Gating strategy	Single cells were gated based onFSC-A and FSC-H features. Live cells were gated based on FVS780 or Live/Dead NIR negative staining. Boundaries of the gates were determined by the visualization of distinct populations or comparing samples andpositive/negative controls.

☒ Tick this box to confirm that a figure exemplifying the gating strategy is provided in the Supplementary Information.
